# Editorial: Ubiquitin and the Brain: Roles of Proteolysis in the Normal and Abnormal Nervous System

**DOI:** 10.3389/fnmol.2017.00220

**Published:** 2017-07-18

**Authors:** Ashok N. Hegde, Fred W. van Leeuwen

**Affiliations:** ^1^Department of Biological and Environmental Sciences, Georgia College and State University Milledgeville, GA, United States; ^2^Department of Neuroscience, Faculty of Health, Medicine and Life Sciences, Maastricht University Maastricht, Netherlands

**Keywords:** proteasome, synaptic plasticity (LTP/LTD), neurodegenerative diseases, Alzheimer disease, Parkinson's disease, Huntington's disease, glutamate receptors, local protein degradation

Proteolysis by the ubiquitin-proteasome pathway (UPP) is now widely recognized as a major molecular mechanism playing a role in numerous normal functions of the nervous system as well as in malfunctions of the brain in several neurodegenerative diseases. In the UPP, attachment of a small protein, ubiquitin, tags the substrates for degradation by a multi-subunit complex called the proteasome. Linkage of ubiquitin to protein substrates is highly specific and occurs through a series of well-orchestrated enzymatic steps. Protein degradation has key functions in the nervous system including fine-tuning of synaptic connections during development and synaptic plasticity in the adult organism (Hegde, 2017). In neurons, several physiological processes are regulated by proteolysis. From gene transcription to posttranslational modification of proteins, several quality checks are essential before the protein is ready for biological action, locally in dendrites or distantly via axonal transport. Beyond regulation at the RNA level, proteolysis by the UPP provides a quick and efficient way to regulate the amount of protein in neurons. In addition, accurate folding and control over levels of many proteins must be tightly regulated both spatially and temporally. To achieve this function, the cell possesses a network of different protein quality control systems (PQC) for protein folding via molecular chaperones as the first line of defense against protein misfolding and aggregation (Ciechanover and Kwon, 2017). Subsequently accurate protein degradation by the UPP and the autophagosomal-lysosomal system is the second line of defense. In addition, recent data suggest an additional PQC pathway in which misfolded proteins are excreted actively after encapsulation at the endoplasmic reticulum, a process dubbed MAPS (Misfolded Associated Protein Secretion) (Lee et al., 2016). It is even speculated that a dysfunctional PQC contributes to the process of proteopathic seeding in Alzheimer's disease (AD) (Gentier and van Leeuwen, 2015). In addition, dysfunction of the UPP is linked to Parkinson's, Huntington's, and other neurodegenerative diseases (Hegde, 2017). Perturbation in the UPP is also believed to play a causative role in mental disorders such as Angelman syndrome (Jiang and Beaudet, 2004).

Many questions pertaining to the UPP in the nervous system remain unanswered. How is the UPP-mediated degradation regulated spatially and temporally in neurons? What is the role of local protein degradation? How does the interplay between proteolysis and protein synthesis affect synaptic plasticity and memory? Do perturbations in the UPP have a role in the pathology of neurodegenerative diseases?

The papers in this *Frontiers in Molecular Neuroscience* Research Topic-Special Issue on *Ubiquitin and the Brain: Roles of Proteolysis in the Normal and Abnormal Nervous System* cover a wide range of topics from development of methods and primary research studies to reviews. These articles from researchers working on yeast, neuronal and glial cell culture and mouse models, often validated in postmortem human brain tissue, cover a wide array of topics such as receptor endocytosis and synaptic plasticity in the normal nervous system to abnormalities of the nervous system such as AD and Huntington's disease.

The paper by Pinto et al. reports the results of studies on visualizing a specific type of ubiquitin linkage to substrate proteins in which ubiquitin molecules are linked to each other through a covalent linkage to Lysine-48 (K48) in the ubiquitin sequence. The K-48 linkage targets substrate proteins for degradation. Pinto et al. study adapted a technique to monitor K-48-liked ubiquitin molecules in cultured rat hippocampal neurons. In this technique, a yellow fluorescent protein called Venus is split into non-fluorescent N- and C- termini and fused to sequences containing ubiquitin-interacting-motifs (UIMs). These parts come together when they bind to closely spaced ubiquitin molecules in a polyubiquitin chain assembled through K-48 linkage. Using this technique, the authors show that proteins tagged with K-48-linked ubiquitin chains accumulate in presynaptic terminals when synapses newly form.

The studies by Scott Wilson and colleagues (Vaden et al.) builds on their previous work on the role of a deubiquitinating (DUB) enzyme called USP14 in the mammalian neuromuscular junction (NMJ). Their studies suggest that the role of USP14 in maintaining the free ubiquitin levels is critical for NMJ structure but the function of NMJ (such as muscle coordination) is likely to be regulated by USP14 through a separate pathway. Apart of USP14, many other DUBs play a role in the nervous system. Ristic et al. argue in their review that looking at the UPP from the point of view of DUBs can provide novel insights into the exquisite regulation of proteolysis in the nervous system and might be helpful in devising therapeutic approaches.

Goo et al. take a look at the role of ubiquitin in regulating trafficking of AMPA-type glutamate receptors. After internalization AMPA receptors can either be recycled back to the plasma membrane or degraded by the lysosome or the proteasome. For example, Nedd4-1, a ubiquitin ligase, is responsible for targeting AMPA receptors to proteasome-mediated degradation. Two deubiquitinating enzymes called Usp8 and Usp46 remove the ubiquitin attachment on AMPA receptors and thus appears to be important for rescuing the receptors from degradation and recycling them back to plasma membrane.

Rodriguez et al. examine the question of increased longevity in female mice with chronic rapamycin treatment and the effect of the drug on proteasome activity and expression of chaperone proteins. They show that rapamycin treatment has different effects on various tissues of the body and increases the amount of proteasome-interacting chaperone proteins in female but not in male mice.

Stojkovic et al. review the key role of ubiquitination in modifying the clock proteins which determine the circadian rhythm. They propose that ubiquitination is a key part of the posttranslational modification “code” that determines the fate and function of a particular clock protein.

Hegde et al. review the accumulated evidence on local roles of protein degradation in induction and maintenance of long-term synaptic plasticity. Because neurons are highly polarized cells, understanding local proteolysis is likely to be important both from a basic science point of view and translational research. Local neuronal function is also emphasized in the mini-review by Zhao et al. with respect to clearance of mutant huntingtin (mHtt) protein. Tagging of mHtt by ubiquitin plays a role in clearance by the proteasome as well as through autophagy. The authors present strategies to study local clearance and accumulation in neuronal sub-compartments.

Jarome and Helmstetter review the evidence for the roles of protein degradation in long-term memory (LTM) storage. They discuss the interplay between protein degradation and protein synthesis in the hippocampus and amygdala and other cortical areas during formation and consolidation of LTM.

The coverage of the role of proteolysis by the UPP in abnormalities of the nervous system ranges from drug abuse to neuroinflammation and neurodegenerative diseases. Massaly et al. review the evidence for role of the UPP in mediating the effects of drug abuse on the nervous system. They details the role of the UPP in regulating molecules that mediate drug-abuse-related neuroplasticity. In addition, they present evidence from the literature regarding how drugs of abuse regulate amounts and function of the various components of the UPP. The review by Figueiredo et al. discusses the role in neuroinflammation of a specific class of prostaglandins called J2 which are the toxic products of cyclooxygenases. Among the pleiotropic effects of J2 prostaglandins is perturbation of UPP. The authors suggest that the targeting neuroinflammatory pathways stimulated by J2 prostaglandins might be beneficial in treating several neurodegenerative diseases such as AD, Parkinson's and amyotrophic lateral sclerosis. Gong et al. discuss the role of components of the UPP such as the deubiquitinating enzyme Uch-L1, F-Box protein Fbx2, and the proteasome in development of AD and spinal cord injury. They suggest pharmacological manipulations of these components could be developed as a therapeutic strategy. In addition, the role of UPP is highlighted in neurodegenerative diseases such as AD, Parkinson's disease and Huntington's disease (papers by Ortega and Lucas; Atkin and Paulson; Jueneman et al.).

Until recently, the investigation with regard to the role of proteolysis in neurodegenerative diseases largely focused on neurons and role of the UPP in glia was largely ignored. This has been true also of other diseases with protein conformational abnormalities such as Amyotrophic Lateral Sclerosis and Parkinson's disease. The review by Jansen et al. tackles this issue and discusses among other things the differences between neurons and glia in how the two cell types react to impaired proteolysis by the UPP.

The studies on the role of the UPP in neurodegenerative diseases can be greatly benefited by work on simple model systems such as yeast. This model system provides an easy readout of perturbations in the UPP and allows quick analysis of action of numerous chemicals on the UPP (see review by Brau), which can be subsequently validated using mammalian model systems. The group of Dantuma has developed many tools to detect proteasomal activity (e.g., constructs with GFP and YFP) *in vitro* as well as *in vivo*. The authors (Dantuma and Bott) discuss “the paradigm shift that has repositioned the UPS from being a prime suspect in the pathophysiology of neurodegeneration to an attractive therapeutic target that can be harnessed to accelerate the clearance of disease-linked proteins” (review by Dantuma and Bott). For example, downregulation misframed ubiquitin (UBB^+1^) that interacts in AD with γ-secretase (Gentier and van Leeuwen, 2015) could be a potential therapeutic target to restore neuronal function perturbed by abnormalities in the UPP. Based on accumulating evidence, it is clear that the neuronal-glial network of the brain functions effectively when protein degradation is normal and impairments in the UPP contribute to the early cellular abnormalities seen in AD.

The research articles and reviews in this research topic now collected as an e-book highlight the complex role of ubiquitin-proteasome-mediated proteolysis in the neuronal and glial physiology and pathology. In spite of the considerable progress made over the last two decades, many challenges remain. For example, with respect to normal roles of the UPP in the nervous system, the roles of the UPP in memory formation especially in relation to the role of protein synthesis need to be better understood. The exact contributions and the interplay of the UPP with other protein-clearance systems such as autophagy in various neurodegenerative diseases also need to be delineated. As many new technological developments such as CRISPR/Cas9, allow precise testing of hypotheses, we may expect to see many additional interesting discoveries on both these fronts.

## Author contributions

Both authors listed have made a substantial, direct and intellectual contribution to the work, and approved it for publication.

### Conflict of interest statement

The authors declare that the research was conducted in the absence of any commercial or financial relationships that could be construed as a potential conflict of interest.
